# Switching Off Key Signaling Survival Molecules to Switch On the Resolution of Inflammation

**DOI:** 10.1155/2014/829851

**Published:** 2014-07-17

**Authors:** Denise Alves Perez, Juliana Priscila Vago, Rayssa Maciel Athayde, Alesandra Corte Reis, Mauro Martins Teixeira, Lirlândia Pires Sousa, Vanessa Pinho

**Affiliations:** ^1^Laboratório de Resolução da Resposta Inflamatória, Departamento de Morfologia, Instituto de Ciências Biológicas, Universidade Federal de Minas Gerais, Avenida Antônio Carlos 6627, Pampulha, 31270-901 Belo Horizonte, MG, Brazil; ^2^Laboratório de Imunofarmacologia, Departamento de Bioquímica e Imunologia, Instituto de Ciências Biológicas, Universidade Federal de Minas Gerais, 31270-901 Belo Horizonte, MG, Brazil; ^3^Laboratório de Sinalização inflamação, Departamento de Análises Clínicas e Toxicológicas, Faculdade de Farmácia, Universidade Federal de Minas Gerais, 31270-901 Belo Horizonte, MG, Brazil

## Abstract

Inflammation is a physiological response of the immune system to injury or infection but may become chronic. In general, inflammation is self-limiting and resolves by activating a termination program named resolution of inflammation. It has been argued that unresolved inflammation may be the basis of a variety of chronic inflammatory diseases. Resolution of inflammation is an active process that is fine-tuned by the production of proresolving mediators and the shutdown of intracellular signaling molecules associated with cytokine production and leukocyte survival. Apoptosis of leukocytes (especially granulocytes) is a key element in the resolution of inflammation and several signaling molecules are thought to be involved in this process. Here, we explore key signaling molecules and some mediators that are crucial regulators of leukocyte survival *in vivo* and that may be targeted for therapeutic purposes in the context of chronic inflammatory diseases.

## 1. Introduction

Inflammation is a reaction of an organism to cell and tissue damage caused by various types of agents (sterile or not, including autoimmune events). Inflammation may also be physiological and is thought to be crucial for the maintenance of tissue homeostasis [1–5]. Important microcirculatory events occur during the inflammatory process, including vascular permeability, changes in the movement, recruitment and accumulation of leukocytes, and the release of inflammatory mediators [6]. After elimination of the harmful agent, the inflammatory process is usually resolved, as seen by the reduction in the number of leukocytes in the inflammatory site and the reversal of vascular changes. Resolution is necessary to restore the original architecture and function of a given tissue. Failure to resolve can cause persistent inflammation with consequent maintenance or increase of tissue destruction [3]. It has been argued that unresolved inflammation or excessive initial inflammation may be the basis of a variety of chronic inflammatory diseases [7].

The resolution of inflammation is an active process that is coordinated and controlled by a variety of extracellular and intracellular molecules [4]. With the termination of the inflammatory stimulus, the reduction of proinflammatory mediators occurs at the site through the decreased synthesis and increased catabolism of these molecules [4, 8]. The release of proresolving mediators also occurs which prevents further migration and increases apoptotic events of leukocytes (primarily granulocytes) [9]. In parallel, some proresolving molecules are able to induce the incoming of nonphlogistic macrophages to further perpetuate efferocytosis of apoptotic granulocytes. In doing so, proresolving molecules reprogram macrophages to perform more restorative and resolutive roles, thus amplifying the production of proresolving molecules and promoting resolution [10, 11]. These events mark the beginning of the resolution process, which is essential to reestablish tissue homeostasis [4, 8, 10, 12].

Some leucocytes, such as granulocytes (mainly neutrophils and eosinophils) and macrophages, are profoundly involved in the inflammatory response. Granulocytes release toxic compounds and also act as phagocytes together with macrophages to remove the agent causing inflammation. However, for the inflammatory process to be successful and self-limiting (with the goal of restoring tissue homeostasis), the actions of neutrophils and eosinophils must be finely regulated [3, 4, 8]. Thus, apoptosis of granulocytes followed by the efferocytosis (phagocytosis of apoptotic cells) by macrophages and an active resolution process are obvious avenues to achieve this goal [6, 10, 12].

**Figure 1:**
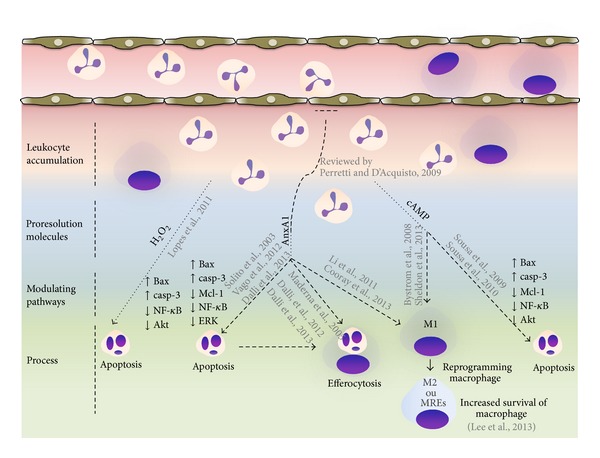
Targets to promote granulocyte apoptosis and inflammation resolution. During early phase of inflammation production of proinflammatory mediators and activation of signal survival pathways (PI3K/Akt, NF-*κ*B, MAPKs, and CDKs) promotes leukocyte accumulation and survival in the inflammatory site. While inflammatory response evolves, local activation/release of proresolution mediators occurs and pathways (H_2_O_2_, AnxA1, and cAMP) that control further granulocyte ingress and turn on a resolution program. These resolution molecules, in addition to proresolving lipid mediators which are not highlighted in this cartoon, downregulate survival pathways and activate an apoptosis-associated program in granulocytes. Resolution molecules are also able to promote efferocytosis and coordinate reprogramming of macrophages. These events will reestablish tissue homeostasis.

Several signaling molecules, including PI3K/Akt, NF-*κ*B, MAPKs, and CDKs, have been shown to be involved in enhancing granulocyte survival* in vivo* and* in vitro* [13–16]. The rationale behind enhanced granulocyte survival involves delaying death of these cells to enable efficient effector function, such as bacterial killing. However, if not finely controlled, prolonged activation of survival pathways and prevention of apoptosis in granulocytes may eventually delay inflammation resolution. In contrast with the molecules described above, proresolving mediators, including Annexin A1 (AnxA1), hydrogen peroxide (H_2_O_2_), cyclic adenosine monophosphate (cAMP), TNF-related apoptosis-inducing ligand (TRAIL) elevating agents (see Figure 1), and other specialized lipid mediators (lipoxin A_4_, resolvins, maresins, and protectins), perform the opposite action; that is, they induce granulocyte apoptosis. Recent studies have shown that strategies that modulate apoptosis-controlling proteins may promote the resolution of the inflammatory process [17–22]. Therefore, potential therapeutic strategies that modulate the resolution pathways may further represent a useful pharmacological arsenal for the treatment and prevention of various acute and chronic inflammatory diseases. Here, we discuss some aspects of the complex signaling network and some interventions that interfere with key signaling molecules associated with leukocyte survival and consequently contribute to inflammation resolution and return to homeostasis.

## 2. Signaling Molecules as Crucial Regulators of the Resolution of Inflammation

### 2.1. Cyclin-Dependent Kinases

Cyclin-dependent kinases (CDK) are serine/threonine protein kinases that bind to cyclin to mediate the phosphorylation reactions that are associated with the progression and regulation of the cell cycle [23]. Surprisingly, terminally differentiated cells, including neutrophils, also express CDKs [24]. Previous studies have demonstrated that human neutrophils express CDK isoforms and their activity is associated with neutrophil lifespan [24–28]. More recent data have shown that CDK inhibitors (CDKi) drive granulocyte apoptosis and resolve inflammation by downregulating Mcl-1 and upregulating proapoptotic proteins such as Bim [24, 26, 27, 29–31]. In several models of inflammation, including passively induced arthritis, bleomycin-induced lung injury, and carrageenan-elicited acute pleurisy, R-roscovitine, a selective inhibitor of cyclin-dependent kinases CDK2, CDK5, CDK7, and CDK9, enhanced the resolution of established inflammation. This resolution was associated with an increase in neutrophil apoptosis in a caspase-dependent manner [24]. In murine models, it has also been reported that the induction of neutrophil apoptosis with R-roscovitine in conjunction with antibiotic therapy reduces markers of neuronal damage of pneumococcal meningitis [30]. Moreover, neutrophil clearance mediated through CDK inhibition reduced the lung inflammation induced by lipoteichoic acid,* Streptococcus* pneumonia, and bleomycin-induced lung injury models [26, 32]. R-roscovitine inhibits the CDK7- and CDK9-dependent phosphorylation of RNA polymerase II to block the transcriptional capacity of neutrophils, which can be a key mechanism associated with neutrophil apoptosis after CDK inhibition [26]. The studies discussed above clearly demonstrate that CDK inhibitors induce apoptosis and* in vivo* clearance of nonproliferating cells, such as granulocytes. AT7519 is a more recently investigated CDK inhibitor that has been evaluated in clinical trials for anticancer therapy [33]. A study by Alessandri et al. demonstrated that AT7519 induced eosinophil apoptosis and enhanced the resolution of allergic pleurisy [31]. AT7519 was also capable of inducing neutrophil apoptosis and accelerating the resolution of inflammation induced by LPS or* Escherichia coli* without the impairment of bacterial clearance [34]. Importantly, AT7519 has already been tested to treat patients with refractory solid tumors [33]. Altogether, these findings suggest that CDK may be a useful therapeutic strategy for the treatment of inflammatory diseases.

### 2.2. Mitogen-Activated Protein Kinases (MAPK) and Extracellular-Signal-Regulated Kinase (ERK)

The mitogen-activated protein kinases (MAPK) represent a family of serine threonine kinases that phosphorylate and activate transcription factors present in the cytoplasm or nucleus to drive the expression of genes and consequently biological responses. There are three main MAPK-activated signaling cascades that lead to differential gene expression: extracellular-signal-regulated kinase ERK1/2, p38 MAP kinase, and c-Jun N-terminal kinases (JNKs); these cascades are activated by several stimuli to regulate proliferation, differentiation, cell survival, mitosis, apoptosis, and other cell functions [35, 36]. The MEK/ERK signaling pathway has been targeted in an attempt to promote resolution of acute inflammation. Thus, it has been demonstrated that treatment with U0126, a potent and selective MEK1/2 inhibitor (a kinase upstream ERK1/2), was able to reduce inflammatory parameters in a murine model of allergic asthma [37] and LPS-induced lung injury [38]. Additionally, the use of a specific ERK1/2 inhibitor (PD98059) augmented the resolution of inflammation in a pleurisy model; this was associated with the inhibition of the production of neutrophil survival factors at the site of inflammation and the increased neutrophil apoptosis [39]. A recent study* in vitro *demonstrated that MSK1/2 (mitogen- and stress-activated protein kinase 1 and 2)—two kinases phosphorylated by both ERK1/2 and p38 MAPK—are associated with control on the induction of cyclooxygenase- (COX-) 2 mRNA and the IL-10 production through CREB (cAMP responsive element-binding protein) in macrophages stimulated or not with LPS [40]. However, there is no evidence that MSK1/2 are relevant for resolution of inflammation* in vivo*. Therefore, although the anti-inflammatory effects of MAPK inhibitors have been clearly defined, the potential of these inhibitors to promote inflammation resolution needs to be better clarified using other models of* in vivo* inflammation.

### 2.3. Cyclic Adenosine Monophosphate

Cyclic adenosine monophosphate (cAMP) is a ubiquitous second messenger produced after adenylate cyclase activation that has been shown to play an important role in modulating the activity of cells involved in the inflammatory process, primarily through PKA activity [41]. Intracellular levels of cAMP are physiologically modulated by agonist ligands (such as PGE_2_, adenosine, and *β*-adrenergic) and are fine-tuned and controlled by phosphodiesterases (PDEs), which are intracellular enzymes that hydrolyze cAMP [41, 42]. In addition to known anti-inflammatory proprieties of cAMP-elevating agents [41–44], emerging data support a role for cAMP in some steps of the resolution process [20, 45–47]. Our research group demonstrated an important role for cAMP in inducing the resolution of acute inflammation by modulating the apoptosis of granulocytes* in vivo *[20, 45]. cAMP elevation, which is mimicked by the administration of cAMP mimetic compounds or promoted by rolipram (a PDE4 inhibitor), induced the resolution of both eosinophilic [45] and neutrophilic inflammation [20], which was mediated by PKA and dependent on granulocyte apoptosis. The resolution induced by increasing cAMP levels has been associated with the modulation of several molecular pathways, which are important for leukocyte survival. For example, it has been shown that high concentrations of cAMP decrease prosurvival intracellular molecules, including MCL-1, PI3K, and NF-*κ*B and increase levels of proapoptotic molecules: BAX and cleaved caspase-3 [20]. Lower levels of cAMP may account for the lack of resolution of inflammation in a murine model of chronic granulomatous disease [48].

Emerging concepts about the role of cAMP in inflammation resolution came first from Bystrom and cols [46], who demonstrated the participation of cAMP in reprogramming inflammatory macrophages to resolution-phase macrophages. In agreement with these findings, a recent paper showed that cAMP contributes to resolution by polarizing M1 to M2 macrophages [49]. In addition to inducing macrophage reprogramming, enhanced levels of cAMP induced by binding to lysophosphatidylserine (lyso-PS) expressed on apoptotic neutrophils are also involved in efferocytosis [50, 51]. Lyso-PS acts on the macrophage G2A receptor and enhances the clearance of these neutrophils by signaling through the PKA-dependent increase of Rac1 activity via an increased production of PGE_2_ and cAMP [52]. However, whether the above-described mechanisms of cAMP may be applied in* in vivo* situations of inflammation remains to be determined.

cAMP may function as an intermediate of the effects of certain proresolutive molecules. For example, one study suggests that RvD1 is able to increase intracellular levels of cAMP and rescue macrophage apoptosis in a cAMP-dependent manner [47]. A recent study by our group demonstrated that treatment with a PDE4 inhibitor, which enhances cAMP, induced resolution of inflammation that was associated with increased levels of AnxA1 [21]. Altogether these data suggest that cAMP is a crucial control molecule in the resolution of inflammation. Not only is cAMP induced by proresolving molecules but it may also induce the release of proresolving molecules, thus acting at multiple regulatory levels to induce resolution. Therefore, cAMP-elevating drugs may represent a useful therapeutic strategy to induce the resolution of inflammation.

### 2.4. Phosphoinositide 3-Kinases

Phosphoinositide 3-kinases (PI3Ks) are a family of intracellular lipid kinases that phosphorylate the 3-OH group of inositol membrane lipids, thus regulating many aspects of cell function, including cell metabolism, survival, and polarity. This family can be divided into three main classes (I, II, and III) based on structural and biochemical characteristics [53, 54]. In mammals, isoforms of PI3K are related to signal transduction, and each isoform plays a different role [53]. Class I is subdivided into two subclasses, IA and IB. PI3K*γ* is the only member of class IB and is the most highly expressed in cells of the immune system. This isoform is composed of the p110*γ* catalytic subunit and the p101 regulatory subunit and is activated by the G*β* *γ* subunits of G proteins [55, 56]. PI3Ks are known to be important in many cell processes related to the immune system, including cell activation, migration [55, 57, 58], and cell survival, via the phosphorylation of Akt/protein kinase B (PBK) [20, 45]; PI3Ks are also activated by antigen, cytokine, and chemokine receptors [59].

Our group has evaluated the role of PI3K*γ* in inducing and maintaining the inflammatory process in experimental models. In a model of allergic pleurisy in mice, the inhibition of PI3K cleared accumulated eosinophils and increased the number of apoptotic events. Experiments using adoptive transfer of bone marrow cells showed that PI3K*γ* on leukocytes was required for the maintenance of eosinophil influx at the later stages of eosinophilic inflammation [17]. These studies suggest that PI3K*γ* on leukocytes is relevant for the maintenance of inflammation and that inhibitors of these enzymes could potentially impart on resolution of inflammation.

However, there are no published studies demonstrating that selective blockade of PI3K*γ* is really proresolutive* in vivo*. Blockers of PI3K*γ* may have anti-inflammatory effects* in vivo,* as we have shown in a model of bleomycin-induced pulmonary inflammation [19]. However, a definite demonstration that these enzymes are relevant* in vivo* is lacking as most published studies have only studied the effects of pan PI3K inhibitors, especially wortmannin and LY294002, in preclinical models of inflammation resolution. There are now many new selective PI3K inhibitors in development [60]. Whether such drugs with greater selectivity and safety profile will resolve inflammation* in vivo* needs to be studied.

### 2.5. Nuclear Factor Kappa B

Nuclear factor kappa B (NF-*κ*B) is a transcription factor that regulates immune response to both injury and infection [61, 62]. NF-*κ*B is a convergence point of several signal transduction pathways by conveying the signals of these molecules to the nucleus and promoting transcriptional activation of genes associated with inflammation and cell survival [62]. The activity of NF-*κ*B is primarily regulated through interactions with inhibitory I*κ*B proteins. The phosphorylation of I*κ*B results in its proteasome degradation and the release of NF-*κ*B (usually composed of p50/p65 heterodimers) for nuclear translocation and activation of gene transcription [61, 62]. Over 750 inhibitors of the NF-*κ*B pathway have been identified, including a variety of natural and synthetic molecules. These molecules act by inhibiting NF-*κ*B nuclear translocation/or transactivation or through I*κ*B super repression [63–67].

Recently, numerous investigations have supported the role of miRNAs in the regulation of NF-*κ*B. miRNAs have been found to be involved in NF-*κ*B signaling by targeting NF-*κ*B regulators and effectors [68, 69]. Recent studies have shown that NF-*κ*B inhibitors may attenuate inflammatory parameters in different experimental models of inflammation [70]. For example, NF-*κ*B inhibitors possess anti-inflammatory effects in models of lipopolysaccharide-induced lung injury [71], traumatic brain injury [72], colitis [73], and pulmonary arterial hypertension [74]. Fewer studies have evaluated the effects of NF-*κ*B inhibitors in the resolution of inflammation. Our research group showed that inhibition of NF-*κ*B promotes resolution in established murine models of neutrophilic and eosinophilic inflammation [22, 45]. The resolution of inflammation induced by NF-*κ*B inhibitors in the models of arthritis [22] and allergic pleurisy [45] was associated with enhanced apoptosis of inflammatory cells.

NF-*κ*B activation usually results in the upregulation of antiapoptotic genes that may lead to cell survival [64]. However, NF-*κ*B may also control genes associated with survival and anti-inflammation [70, 75]. In this sense, a few studies have shown that NF-*κ*B inhibitors failed to promote the resolution of inflammation [20] and actually prolonged the inflammatory response by preventing leukocyte apoptosis [70, 76]. Greten and cols also described that NF-*κ*B could also function as a negative posttranslational regulator of inflammasome activation. Therefore, it is clear that NF-*κ*B may have dual role, both pro- and antiresolution, in models of inflammation. This duality of function of NF-*κ*B is likely the result of the central role of this molecule in the convergence of several inflammatory signals [70]. Whether manipulating NF-*κ*B in inflammation will ultimately result in beneficial functions clearly deserves further investigation.

The discussion above suggests that several signaling pathways have been implicated in leukocyte survival during inflammatory response. It is important to note that each specific molecule associated with a signaling pathway cascade may not work in a disconnected manner. Crosstalk between signaling pathways is likely to be essential for leukocyte survival and much more work is needed to understand the interaction between potential resolution inducing pathways, especially in the context of the complex* in vivo* situation of an inflammatory response. However, as demonstrated above, there are molecules which are crucial for resolution of inflammation and whose effects may be indeed exploited therapeutically [77, 78].

## 3. Molecules Involved in Apoptosis of Granulocytes and Resolution of the Inflammatory Response

### 3.1. Annexin A1

Annexin A1 (AnxA1) is a 37 kDa glucocorticoid-induced protein firstly identified by its action on phospholipase- (PL-) A2 inhibition and prevention of eicosanoid synthesis [79]. AnxA1 is a protein member of the annexin superfamily, which exerts its anti-inflammatory activity by binding to receptor ALX (named FPR2, formyl peptide receptor-2, murine), which is also shared with lipoxins [80]. During the initial steps of acute inflammation, AnxA1 limits the recruitment of leukocytes and the production of proinflammatory mediators [80]. During the resolution phase, AnxA1 acts by inducing the apoptosis of neutrophils and this effect is associated with increased expression of cleaved caspase-3 and BAX and decreased expression of pERK1/2, NF-*κ*B, and MCL-1 [21, 81–83] and increasing efferocytosis by macrophages [83–85]. Interestingly, activation of FPR2 by AnxA1 and LXA4 skewed M1 macrophages to M2-like cells [86]. In this context of macrophage modulation, it was demonstrated that AnxA1 released from apoptotic cells contributed to the immunomodulatory effect of these cells on inflammation cells by damping inflammatory monocyte activation [87]. Additionally, AnxA1 may induce indirectly the chemoattraction of monocytes [88]. These effects—migration of monocytes and skewing towards a M2-like phenotype—may be relevant in the context of inflammation but remain to be determined* in vivo*.

The N-terminal region of AnxA1 is the major effector portion responsible for the anti-inflammatory action of the protein [80]. However, once in the extravascular space, the major part of the active AnxA1 (37 kDa) contained in neutrophils is cleaved by proteases, particularly elastase and proteinase-3, yielding the inactive AnxA1 (of 33 kDa form) [89, 90]. As a strategy to deliver anxA1* in vivo*, we and others have explored the therapeutic potential of an AnxA1 peptidomimetic Ac2-26, which retains the biological activity of the entire protein. In a model of acute inflammation triggered by LPS, the administration of Ac2-26 at the peak of inflammation resolved inflammation by inducing caspase-dependent apoptosis of inflammatory cells [21]; this mechanism was also demonstrated using a longer peptide, AnxA1_2–50_ [83]. An AnxA1 cleavage-resistant protein and an AnxA1_2–50_ peptide with a mutation on the cleavage site were demonstrated to be more effective in improving several parameters of inflammation compared with a full length protein and a peptide that was not mutated at the sites of proteases action [83, 90].

One intriguing characteristic of the FPR receptor is that it recognizes both proinflammatory and proresolving signals, thus integrating contrasting cues to determine the course of inflammation [91, 92]. Cooray and cols revealed this intriguing receptor characteristic and showed that the anti-inflammatory signal triggered by AnxA1 (Ac2-26 peptide) and LXA4 promotes FPR2 homodimerization and resolution activities by inducing p38-induced IL-10 production; this stands in contrast with proinflammatory signals, such as SAA, that bind to the receptor alone. Interestingly, Ac2-26 peptide, which is a nonselective FPR ligand (binding to both FPR2 and FPR1), is able to promote the dimerization of FPR1 and FPR2 and change the proinflammatory nature of FPR1 by transducing JNK/caspase-3 proapoptotic signal and promoting resolution of inflammation [93]. These latter findings would help explain the restorative role of Ac2-26 peptide by acting through FPR1 and orchestrating epithelial repair in a model of mucosal inflammation [94]. Thus, AnxA1, its peptidomimetics, or AnxA1-inducer drugs may have great therapeutic potential as resolution inducing drugs* in vivo*.

### 3.2. Hydrogen Peroxide

The nicotinamide adenine dinucleotide phosphate oxidase (NADPH oxidase) expressed in phagocytes is a multisubunit enzyme complex that generates hydrogen peroxide (H_2_O_2_) and other reactive oxygen species (ROS) [95]. Accumulating data has suggested that ROS are not merely injurious but can also downregulate inflammation and contribute to the maintenance of tissue homeostasis [96, 97]. Consistent with this observation, various lines of evidence have indicated a critical role of H_2_O_2_ for the natural resolution of inflammation and regeneration/repair of tissue by inducing apoptosis in different cell types such as neutrophil [22], hepatocyte [98], myocytes [97], and endothelial [99] and lymphoma cells [100].

Our group has investigated the effects of H_2_O_2_ in the context of the resolution of inflammation. Lopes et al. demonstrated that H_2_O_2_ resolves neutrophilic inflammation by activating BAX and caspase-3 and the shutting down NF-*κ*B and PI3K pathways. Consistently with the latter observation, deficiency of the gp91^phox^ component of NADPH oxidase was associated with increased inflammation in a model of antigen-induced arthritis.* In vitro*, H_2_O_2_ has been shown to induce programmed cell death in various cell types, including leukocytes. In these cells, H_2_O_2_ appears to decrease survival signaling pathways, including PI3K/Akt, the transcription factor NF-*κ*B, and mitochondrial pathways [22, 101–104]. The situation* in vivo* is much less well known and studies will be needed to determine the precise molecular pathways that control H_2_O_2_ production and the extracellular and intracellular signaling mechanisms through which H_2_O_2_ promotes resolution of inflammation. In this sense, a recent study showing that H_2_O_2_ may induce the expression of AnxA1 raises the hypothesis that AnxA1 could be involved in the proresolving abilities of this molecule [105]. Therefore, although the proresolving role of H_2_O_2_ is of great interest, further studies on its source and mechanisms of action are clearly needed.

### 3.3. TNF-Related Apoptosis-Inducing Ligand

The TNF-related apoptosis-inducing ligand (TRAIL) is a cytokine that belongs to the TNF superfamily that was discovered in 1995 [106–108]. TRAIL is able to interact with two proapoptotic death receptors, TRAIL-R1/DR4 and TRAIL-R2/DR5, as well as three decoy receptors without functional death domains [109, 110]. The role of TRAIL in biological systems is complex. Several studies have demonstrated a key role of TRAIL in controlling a number of different types of cancer [109–115]. However, some studies have shown that TRAIL has important functions in the immune system, including an immunoregulatory function [116–120]. TRAIL is also involved in the control of some autoimmune diseases [121, 122]. For example, the neutralization of endogenous TRAIL may prevent the resolution of granulomatous experimental autoimmune thyroiditis [123].

A few studies have demonstrated that TRAIL is able to promote apoptosis of human and murine neutrophils [108, 124] and may, hence, promote inflammation resolution. In this regard, it has been shown that the duration of neutrophilic inflammation is enhanced in TRAIL-deficient mice [108]. In addition, TRAIL-deficient mice showed an aberrant inflammatory response associated with reduced apoptosis of inflammatory cells and increased collagen deposition in a model of chronic pulmonary inflammation induced by bleomycin [125]. TRAIL was also found to modulate allergic inflammation. The treatment with antiTRAIL antibody blocked apoptosis of T helper type 2 (Th2) cells and eosinophils and enhanced the inflammatory response [126]. Although conclusive evidence is lacking for a role of TRAIL in the resolution of inflammation [127], the effects of TRAIL are associated with the apoptosis of leukocytes, suggesting TRAIL's potential therapeutic use for the treatment of established inflammatory diseases. Agonistic antibodies have been produced to treat many cancers; the monoclonal antiTRAIL-R1 antibody (mapatumumab) [128] is currently in clinical development, and five antiTRAIL-R2 antibodies are also being tested (lexatumumab, Apomab, TRA-8, AMG 655, and LBY135) [129–133]. Thus, these antibodies could potentially be explored in the context of inflammation resolution.

## 4. Concluding Remarks

Given the importance of inflammation and its resolution, many studies have sought to better understand the molecular scenario involved in these processes. Some of the actors involved in the resolution of inflammation were mentioned in the present review and were demonstrated to be potential targets of therapeutic approaches. The resolution of inflammation is a vital process that enables the return to homeostasis of the immune system and the organ affected by inflammation, avoiding the development of chronic and autoimmune diseases [8, 134].* In vitro* studies provide essential information about molecular machinery that helps to elucidate how different intracellular molecules control leukocyte survival in inflammatory sites. However, they do not cover the entire complexity of* in vivo* settings, which include intracellular pathways and molecules that are interrelated or codependent [78]. Moreover, resolution of inflammation, an* in vivo* phenomenon, is much more complex than simple apoptosis of leukocytes and includes switching off proinflammatory pathways, efferocytosis, and the function of cells other than leukocytes. For example, some proresolving mediators act in macrophages by rescuing from apoptosis and by activating them to induce phagocytosis of apoptotic leukocytes (efferocytosis) [53]. There is also evidence to suggest that nonhematopoietic cells may be involved in the context of the resolution of inflammation [135]. However, studies evaluating the role of epithelial cells and other nonprofessional phagocytic cells in the resolution of inflammation are lacking.

Future studies should try to integrate current findings with single signaling molecules with more complex signaling pathways and how they interact with each other, all of these in the complex* in vivo* situation. Consideration should be given not only to pathways associated with apoptosis of leukocytes but also to molecules and pathways associated with triggering efferocytosis. One must also keep in mind that cells other than leukocytes may respond to resolution inducing molecules* in vivo* by releasing secondary mediators which themselves could be more relevant as final mediators of resolution. New animal models in which natural resolution of inflammation does not occur are clearly needed, especially in models accompanied by a degree of chronic fibrosis. Most reported studies have been performed in systems in which resolution eventually occurs. Whether strategies which speed the resolution of inflammation will also resolve inflammation and prevent or reverse fibrosis in a nonresolving chronic model needs to be determined. Finally, one will also need to take the difficult task of translating experimental findings into human diseases. Whether proresolving strategies will be accompanied by significant degree of immunosuppression is currently not known and will need to be addressed in the future.
